# A Degrading Potassium Tablet Mimicking Active Gastric Bleeding in a Computer Tomographic Investigation

**DOI:** 10.1155/2020/9791519

**Published:** 2020-03-18

**Authors:** J. P. Commandeur, A. Metwaly, L. Büchler, J. Speiser, L. Brander, A. Reintam Blaser

**Affiliations:** ^1^Department of General Surgery, Luzern Canton Hospital, Spitalstrasse, 6000 Luzern 16, Switzerland; ^2^Intensive Care Unit, Luzern Canton Hospital, Spitalstrasse, 6000 Luzern 16, Switzerland; ^3^Department of Anesthesia, Luzern Canton Hospital, Spitalstrasse, 6000 Luzern 16, Switzerland; ^4^Department of Radiology, Luzern Canton Hospital, Spitalstrasse, 6000 Luzern 16, Switzerland

## Abstract

A 54-year-old male patient was admitted to the hospital due to symptoms caused by an intramural hematoma of the descending aorta. In a contrast media-enhanced computed tomography scan performed five days after admission to evaluate dynamics of the hematoma, a hyperdense lesion was seen in the stomach. A suspicion of gastric hemorrhage was raised at the first evaluation. Because the patient's clinical condition and hemoglobin levels were stable, gastroscopy to rule out an aorto-gastric fistula or another type of bleeding was not undertaken. In the secondary evaluation of the history and images, it became clear that the hyperdense lesion mimicking bleeding in the stomach must have been caused by a degrading potassium tablet ingested by the patient five hours before the investigation.

## 1. Introduction

Computed tomography (CT) has become one of the diagnostics of choice for many pathologies, including vascular lesions and detection of active bleeding. Demonstration of extravascular contrast media may indicate active bleeding and should trigger prompt evaluation and potentially further investigations or interventions (e.g., angiography, endoscopy). As described in a recent study, potassium tablets are highly radiopaque [1]. Surprisingly little information is available on potential confusion of radiopaque tablets with extravasal contrast media.

In this case report, we present a case where radiopacity of a degrading potassium tablet in the stomach was first misinterpreted as an extravasation of contrast media related to a bleeding in the stomach. Additionally, we summarize the available literature.

## 2. Case Presentation

A 54-year-old male patient suddenly developed strong thoracic pain and recurrent dysesthesia of both thighs. The emergency medical physician measured a blood pressure difference on the upper extremities of 50 mmHg. Furthermore, intermittently, the pulsations of the bilateral femoral arteries were not palpable. Otherwise, vital signs were normal. With the suspect of an aortic dissection, the patient was immediately transported to the hospital by helicopter. A CT angiography revealed a long-distance intramural hematoma of the descending aorta. For monitoring and strict blood pressure control, the patient was admitted to our ICU (intensive care unit).

**Figure 1:**
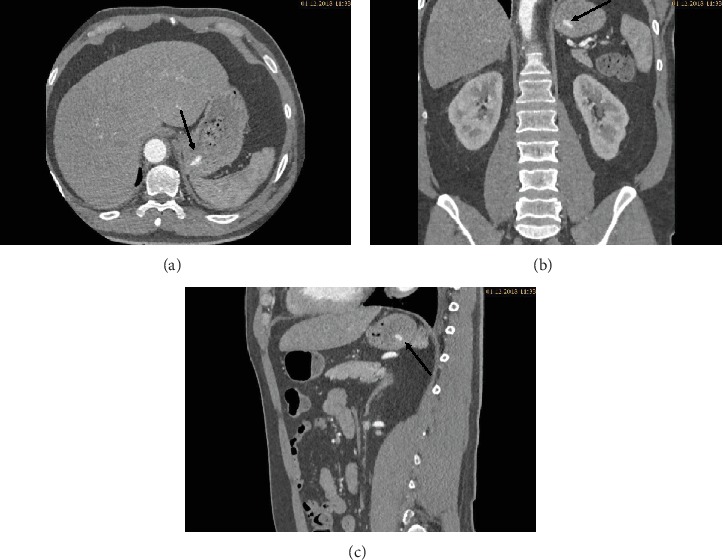
(a–c) Transversal, coronal, and sagittal slides of the abdominal CT in the arterial phase: a faint hyperdense lesion is seen in the posterior fundus of the stomach.

**Figure 2:**
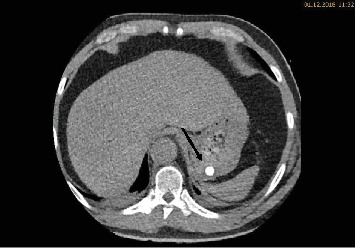
Transversal slide of the abdominal CT in the arterial phase: a sharply demarcated structure in the stomach is seen.

**Figure 3:**
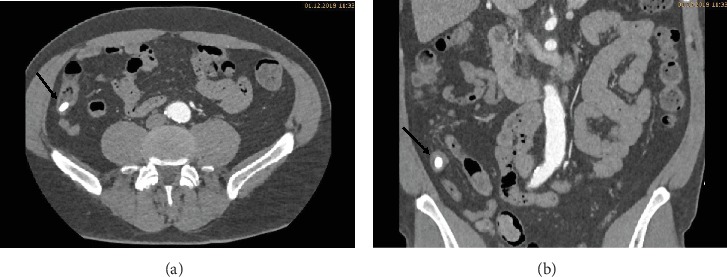
(a, b) Transversal and coronal slides of the abdominal CT in the arterial phase: a sharply demarcated structure in the small bowel is seen.

During the ICU stay, the patient's condition was stable. Bed rest was prescribed, and strict blood pressure control was performed with continuous intravenous infusion of labetalol and urapidil targeting a systolic blood pressure below 120 mmHg. Oral antihypertensive treatment with nebivolol, moxonidine, and amlodipine was started. Continuous labetalol and urapidil infusions were stopped on day three. Due to hypokalemia, potassium chloride tablets were prescribed. Five days after admission, another CT angiography was performed to review the development of the hematoma. The CT scan revealed a reduction of the hematoma. Furthermore, in the arterial phase, a faint hyperdense lesion (max. 348 HU (Hounsfield units), 16 × 8 mm) was detected in the posterior fundus of the stomach (Figures 1(a)–1(c)). The previously acquired native scan was performed only for the thorax and did not include the stomach; therefore, it was not possible to determine with certainty whether the lesion reflected contrast media extravasation or foreign material. However, due to the intramural hematoma of the descending aorta, the possibility of an aorto-gastric fistula was considered. Additional measurements of hemoglobin were performed to screen for relevant bleeding. As the patient's hemodynamics as well as hemoglobin levels remained stable, no further investigations (e.g., gastroscopy) were undertaken and our strategy remained expectative. At the same time, we were searching for an alternative clarification of the findings. We found that (1) the patient had ingested two potassium chloride tablets five hours before the CT scan, (2) potassium chloride tablets have radiopaque properties [1, 2], and (3) there were other hyperdense but sharply demarcated structures in the stomach and in the small bowel (density max. 1724 HU) (Figures 2, 3(a), and 3(b)). After putting all available information together, we suggested that the radiopaque lesion in the stomach was caused by the remnants of a degrading potassium chloride tablet ingested by the patient hours before the CT investigation whereas the other potassium chloride tablet ingested at the same time had been propulsed to the small bowel without being degraded in the stomach.

After eleven days in stable condition, the patient was discharged from the hospital with blood pressure regulating medication.

## 3. Discussion

We describe a case where a degrading potassium chloride tablet mimicked extravasation of contrast media in CT angiography and misled to a suspicion of active bleeding in the stomach.

The fact that potassium tablets are highly radiopaque and under certain circumstances may mimic contrast agent on radiograph images is not widely recognized in everyday clinical practice. There are no specific rules to restrict their administration before imaging such as CT angiography.

The radiopacity of ingested medications was first described in 1987 in the Annals of Emergency Medicine, where the authors examined the radiopacity of 312 different pills. They categorized radiopacity as absent, strong, or weak at the greatest depth of visibility. 22 of the studied pills turned out to be significantly radiopaque; iron-containing compounds were the most radiopaque ones; potassium and calcium carbonate tablets showed a significant radiopacity too [2].

Recently, Sieron et al. measured the radiopacity of the 50 most frequently used medications by CT scan and observed the second highest density for potassium tablets. At 120 kV (kilovoltage), they measured a density of 2829 HU. Cordarone, Concor, Aldactone and Lisinopril complete the top five measured tablets with the highest density [1].

After thorough search of the existing literature, we identified only three case reports where hyperdense lesions caused by ingested pills led to confusion in the therapy.

In 1998, Florez et al. published a case report of a 71-year-old woman who was diagnosed with cholecystolithiasis. In the plain X-ray abdomen, her gall bladder appeared full of stones which were interpreted as gall stones. In a follow-up abdominal X-ray, the stones where located in the mid abdomen. During gastroscopy, their location in the stomach was confirmed. The “stones” turned out to be iron tablets [3].

In an 8-year-old girl and an 8-year-old boy, both treated for (prevention of) recurrent urolithiasis, potassium tablets were reported to be probably responsible for causing a stone-like image confounding with a stone in the kidney region. In both cases, repeated images in various positions showed moving of these radiopaque lesions and the additional history revealed a previous ingestion of potassium tablets. Unnecessary investigations/interventions could be avoided in both children [4, 5].

In all these described cases, a stone-like lesion rather than a barb or a cloud was observed, whereas we did not identify any case describing that a degrading tablet might mimic extravasation of contrast media.

In our case, the initial suspicion of gastric hemorrhage was supported by the underlying disease but not by clinical and laboratory observations. Based on the absence of respective clinical and laboratory findings, as well as on careful reassessment and interpretation of radiological findings, we refrained from performing immediate gastroscopy. Performing a gastrointestinal endoscopy is not without risks. Possible complications include general complications related to sedation and specific complications related to diagnostic and therapeutic maneuvers (e.g., perforation) [6]. The overall complication rate of upper gastrointestinal endoscopy is reported below 1% [6, 7], but the incidence of unplanned events related to gastrointestinal endoscopy under sedation is reported as high as 23% [8].

In general, before performing an angiography CT, a CT without contrast agent is performed. In our case, this investigation was performed as well but only cranial of the hyperdense lesion since the intention of the scan was to evaluate the aortic hematoma. Therefore, we could not differentiate whether the lesion was present already before the application of contrast agent and hence exclude bleeding. In clinical practice, another way to differentiate between a bleeding and another hyperdense structure is the measurement of the density (HU). Intraluminal bleeding with appearance of intravenous contrast media usually measures density of approximately 100-600 HU [9], whereas metals and foreign bodies commonly are of much higher density (e.g., cement 1423 HU, aluminium 2329 HU) [10]. However, this measurement may appear less useful in the case if initially a very high-density tablet has degraded and diluted within the luminal contents as described in our patient, as the maximal measured density of the lesion was 348 HU.

To our knowledge, this is the first case report describing confusion in diagnosis caused by the radiopaque properties of a potassium tablet mimicking bleeding. Careful assessment and interpretation of all available findings were crucial in this case to refrain from an unnecessary gastroscopy.

Potassium supplementation is frequently performed in ICU patients as many of them suffer from electrolyte disturbances in the course of their disease.

In the light of our case, restriction or at least careful documentation of oral medication with radiopaque properties before a scheduled CT scan should be considered to increase patient safety. Such a procedure may avoid confusion in interpretation of the radiographic findings as well as prevent unnecessary investigations or interventions.

## 4. Conclusion

When observing hyperdense lesions in the gastrointestinal system without a strong suspicion of bleeding based on clinical and laboratory findings, one should be aware of radiopaque medication such as, e.g., residues of a potassium tablet.
